# Biomechanical Profiles of Diagnosed Concussions in Rugby Union: A Case Study Using Instrumented Mouthguards

**DOI:** 10.1186/s40798-026-01068-z

**Published:** 2026-07-13

**Authors:** Jonathan Ward, James Tooby, Damien Bonnet, Mickael Roumeau, Loic Louit, Patrice Halimi, Jean-Jacques Raymond, Julien Piscione, Olivier Gavarry

**Affiliations:** 1 Provence Rugby, Aix en Provence, France; 2 Youth Laboratory - Physical Activity and Sports - Health (J-AP2S), Toulon University, Toulon, France; 3 Carnegie Applied Rugby Research (CARR) Centre, Carnegie School of Sport, Leeds Beckett University, Leeds, UK; 4 Aviron Bayonnais, Bayonne, France; 5 French Federation of Rugby, Marcoussis, France; 6 Sport Medicine and Traumatology Unit, St Musse Hospital, CHITS, Toulon, France; 7 South Rugby League Medical Commission, FFR Le Pradet, Marseille, France

## Abstract

**Background:**

This case study presents biomechanical profiles of three confirmed concussions in professional Under 21 (U21) academy rugby union players, recorded using instrumented mouthguards (iMGs).

**Methods:**

Across 34 matches (254 player matches) and 241 playing hours, 4414 head acceleration events (HAEs) were captured, with three concussions clinically diagnosed. Metrics analysed included Peak linear acceleration (PLA), Peak angular acceleration (PAA), Peak angular velocity (PAV), and angular measures: change in Peak angular velocity (ΔPAV) and Rotational Velocity Change Index (RVCI).

**Results:**

Peak linear acceleration ranged from 27.6 to 48.4 *g*, PAA from 3234 to 4089 rad/s^2^, and ΔPAV from 17.7 to 28.5 rad/s. Player #3, who lost consciousness following a high-speed head-on-head collision, recorded the highest ΔPAV (28.5 rad/s) and RVCI (27.3). Player #2, diagnosed post-match, showed the highest cumulative angular load (ΔPAV: 144.3 rad/s; PAA: 17,340 rad/s^2^) despite no clear concussive impact, suggesting cumulative sub-concussive exposure.

**Conclusion:**

These findings demonstrate that concussions can result from both single high magnitude impacts and cumulative impacts. The variability in biomechanical signatures reinforces the need for multi-metric monitoring approaches rather than reliance on isolated thresholds. This study is the first in rugby to report confirmed concussions using iMGs with both peak and cumulative kinematic data. The results support the integration of angular metrics and cumulative load monitoring to understand concussion risk and management in collision sports.

## Key Points

This case study examined three medically confirmed concussions in professional academy rugby players, using instrumented mouthguards to measure head acceleration events during match play.

- One player experienced immediate loss of consciousness following a single, high-impact collision that produced the highest kinematic magnitude recorded in the study.
- The other two players were diagnosed with concussions after the match, and post-game analysis revealed both had sustained multiple head impacts within a short timeframe, with the second impact classified as a severe HAE, suggesting that closely spaced impacts may have contributed to the injury.

These findings indicate that concussion risk arise not only from one-off, high magnitude impacts but also from the cumulative effect of repeated impacts in quick succession. This highlights the importance of considering both impact severity and timing when assessing head impact exposure and developing strategies for player safety.

## Introduction

In collision sports the identification and quantification of head acceleration events (HAEs) have become crucial in understanding both concussive and sub-concussive impacts [1], especially since previous research has found that former international rugby players had a significantly higher risk of developing neurodegenerative diseases compared to the general population [2]. An HAE is defined as an event where linear and/or angular head accelerations are recorded [1]. Instrumented mouthguards (iMGs) have emerged as a validated tool [3] to measure HAEs, offering insights into player exposure. While iMGs have been implemented in American football [4–6], ice hockey [7, 8], and combat sports [9, 10], their use in rugby has recently expanded due to World Rugby mandating their use in all professional competitions from 2024.

Previous rugby union research employing iMGs has primarily examined HAEs in senior professional and amateur men’s and women’s rugby [11–13]. However, there remains a notable gap in the literature concerning professional academy-level players, the developmental stage bridging youth and elite senior competition, where contact intensifies but remains under-characterised.

To the authors knowledge, no research has been reported on case-level analysis of confirmed sports related concussions (SRC) captured via iMGs in professional Under 21 (U21) Academy Rugby Union. A SRC is a traumatic brain injury typically resulting in a rapid onset of short-lived neurological impairment that resolves spontaneously, although in some cases symptoms may evolve over time [14]. Case studies of athletes diagnosed with a concussion can help identify the biomechanical patterns associated with concussive events. Further, these data offer a crucial bridge between biomechanical measurements and clinical diagnosis, which is essential for developing accurate, real-world concussion detection systems.

This case study presents the biomechanical profiles of three confirmed concussions recorded using iMGs in French professional U21 academy rugby. By comparing peak and cumulative match HAE values, we aim to describe the kinematic characteristics of concussive impacts, draw parallels to existing literature, and contribute to a growing body of evidence informing HAE thresholds for clinical relevance in rugby union.

## Methods

### Study Design and Participants

This prospective observational case study was conducted over two competitive seasons and involved 32 male athletes from a French professional rugby academy (Under-21). A total of 4414 HAEs were recorded across 34 matches (254 player matches) representing a cumulative playing time of 241 h. All players were fitted with custom-made, via digital dental scan, iMGs (Prevent Biometrics, USA), which captured real-time linear and angular kinematic data during match play. Only HAEs recorded during official competition matches were included in the analysis. Matches were recorded using a Camera (Sony Handycam HDR-PJ410, Tokyo, Japan), mounted at a height of 4 m on the halfway line of the rugby field, and utilised by the video analyst. The footage was subsequently analysed to confirm and identify the match action associated with the concussive HAEs.

### Instrumented Mouthguards and Head Acceleration Severity Measures

Three kinematic metrics were recorded using iMGs, with data recording triggered when acceleration on any single axis exceeded 8 *g*. Although the trigger threshold is set at 8 *g* based on measurements at the iMG sensor, HAEs with magnitudes below 8 *g* can still be captured. The values are calculated after transforming the kinematic data to the head’s CoG, with the peak values based on the resultant of all three axes.

Peak linear acceleration (PLA, *g*), Peak angular acceleration (PAA, rad/s^2^), and Peak angular velocity (PAV, rad/s). Peak linear acceleration was measured by the tri-axial accelerometer, while PAV was measured using a gyroscope. Peak angular acceleration was derived through numerical differentiation of the angular velocity signal, as described in previous research [15].

To minimise false positives, a combined recording threshold of 5 *g* (PLA) and 400 rad/s^2^ (PAA) was applied after data transformation in accordance with prior research [11] to exclude low-magnitude single axis non-contact events (e.g., running, jumping). 4414 HAEs exceeding both thresholds and were included in the analysis.

Head Acceleration Events were approximated indirectly by capturing Sensor Acceleration Events (SAEs), which are brief periods of iMG sensor measurements processed to estimate head kinematics. An SAE is triggered when the iMG sensor exceeds a predefined threshold, set at 5 *g* for this study. Once triggered, the iMG records 10 ms of pre- and 40 ms of post-trigger data. The raw kinematic signals are first filtered to eliminate electrical noise using a zero-phase, 4-pole Butterworth low-pass filter. Cut-off frequencies were applied based on signal quality: 200 Hz for minimal noise, 100 Hz for moderate, and 50 Hz for severe. A proprietary algorithm classified signal quality into three levels: Class 0 (minimal noise), Class 1 (moderate), and Class 2 (severe). Following filtering, linear kinematic data were transformed from the iMG location to the head’s centre of gravity (CoG) to provide a more accurate representation of head motion.

The iMGs reliance on a preset trigger threshold and a short re-arming period, during which subsequent impacts cannot be recorded, may have led to missed HAEs, particularly for sub-threshold impacts or those occurring in rapid succession [15].

### Statistical Analysis

**Fig. 1:**
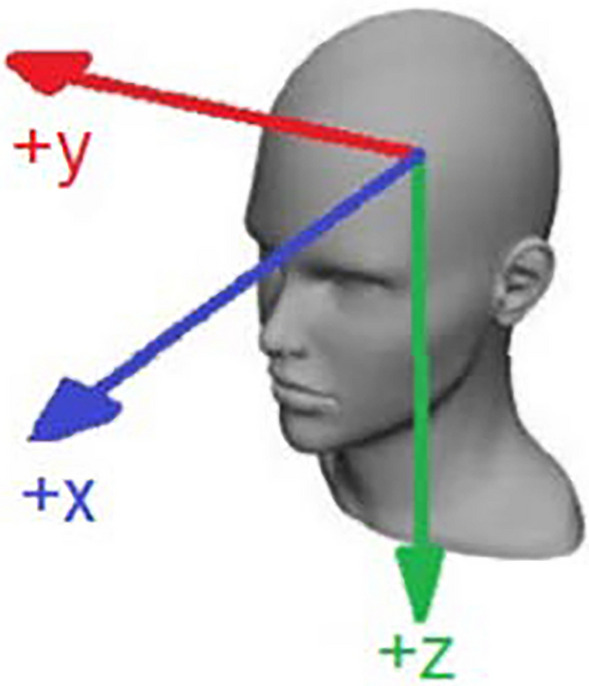
Axes of head kinematics relative to the head centre of gravity

Cumulative loading was calculated by summing all recorded HAEs for each player. Two additional metrics were computed: ΔPAV (change in angular velocity, rad/s) and the Rotational Velocity Change Index (RVCI, rad/s). The rationale for including RVCI in the analysis is due to the growing recognition that angular kinematics better represent brain injury mechanisms than linear acceleration alone [16]. ΔPAV was calculated as the maximum resultant angular velocity change following zero-referencing of each component axis (X, Y, Z), as illustrated in Fig. 1, to the pre-impact baseline of each HAE. This approach captures the maximum change in angular velocity across all axes during an HAE. RVCI was computed using a custom MATLAB script based on the method described by Yanaoka et al. [17] in which the maximum resultant angular velocity change is identified across a sliding time window constrained to 10 ms. The calculation incorporates axis-specific weighting factors which were selected to optimise correlation with Maximum Principal Strain in finite element brain models. As shown in research by Zhan et al. [18], RVCI is a composite measure that quantifies how much and how suddenly the head’s angular velocity changes during an impact. It reflects the overall angular loading on the brain by combining angular velocity changes across all three axes, with weighting factors that account for the brain’s sensitivity to rotation in different directions. Higher RVCI values indicate greater angular stress, which is closely linked to brain strain and increased concussion risk [18].

## Results

During the study, eight concussions were diagnosed within the team, three of which occurred while athletes were wearing their iMGs. Players #1 and #2 remained asymptomatic during match play but were diagnosed post-game following clinical assessment. It was not possible to determine the specific match action that led to potential concussive symptoms, such as lying on the ground after contact, stumbling upon rising, or holding the head, based on the video footage. As a result, we are unable to provide insight into the exact game events responsible for the concussions for Players #1 and #2. Player #3 experienced an immediate loss of consciousness following a head-on-head collision, as observed in the video footage. The incident occurred as the player was sprinting downfield while tracking an up-and-under kick (also referred to as a high ball), and their focus was on the trajectory of the ball, not on the opposition player who was running forward to contest the ball. The ball was caught by an opposing player who was slightly to the left of Player #3. The resulting head-to-head impact produced a rapid displacement of Player #3’s head toward the left (transverse plane rotation). There was no evident effort to lower body height, wrap the arms, or initiate contact using the shoulder. The iMG recorded the location of the HAE as “BOTTOM_FRONT”.

**Fig. 2:**
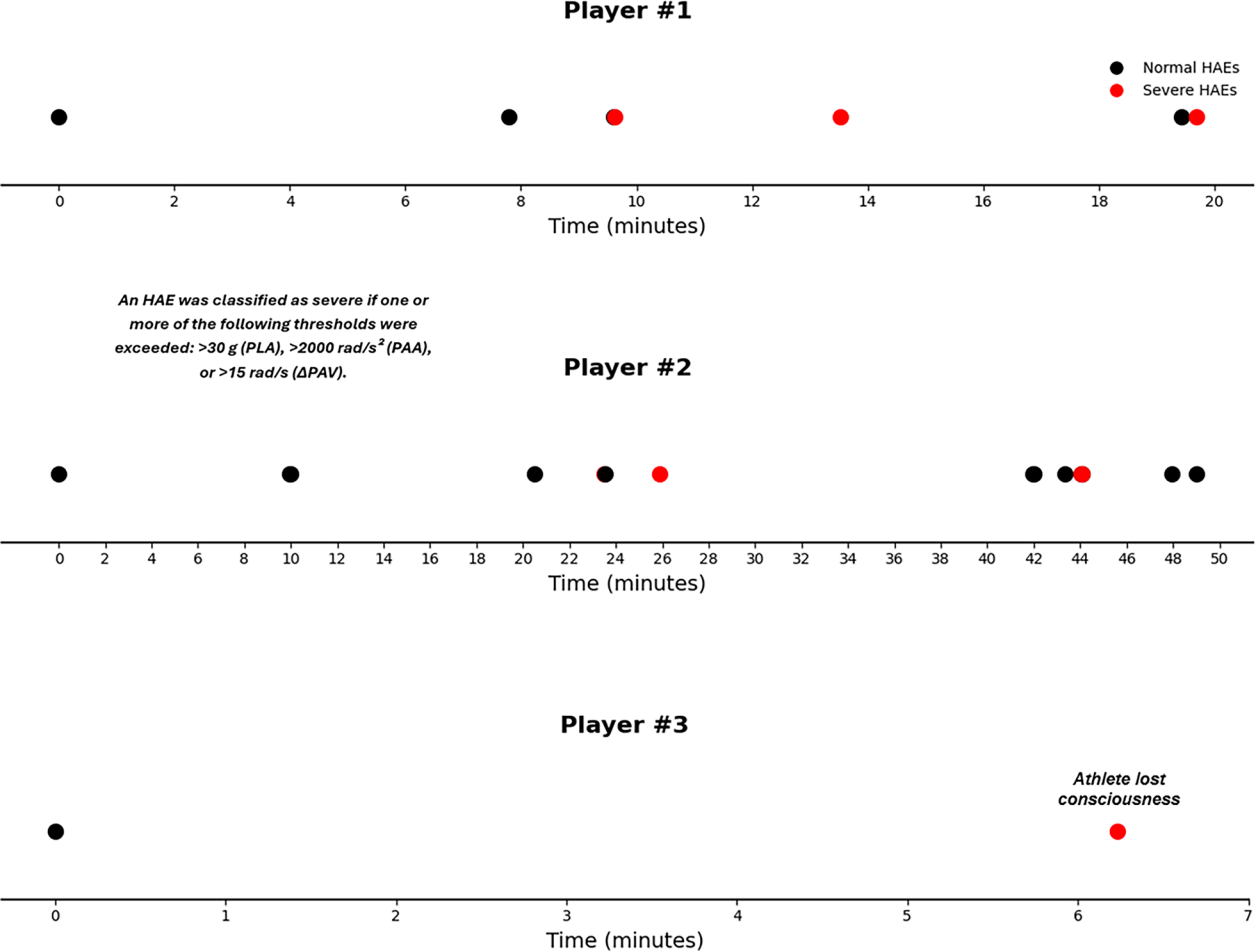
Timeline of HAEs across the three players during match play that lead to concussion Diagnoses. The first HAE for each player was set at 0 min. A delay in player 2's timeline corresponds to a 10-min stoppage due to an injury to another player, explaining the gap in HAEs during this period

Maximum PLA values ranged from 27.6 to 48.4 *g*, PAA from 3234 to 4089 rad/s^2^, and ΔPAV from 17.7 to 28.5 rad/s. Notably, Player #3 exhibited the highest ΔPAV and RVCI (28.5 rad/s and 27.3 respectively). Cumulative loading varied between players, with Player #2 accumulating the greatest total angular burden across the match (ΔPAV:144.3 rad/s; cumulative PAA 17340 rad/s^2^). The biomechanical characteristics of these three events are summarised in Table 1, with Fig. 2 showing the distribution of HAEs across a match for each player. Each dot represents a single HAE, with red markers indicating events classified as severe (i.e., exceeding > 30 g for PLA, > 2000 rad/s^2^ for PAA, or > 15 rad/s for ΔPAV), and black markers representing non-severe events. This figure illustrates the temporal variability of HAEs and highlights the occurrence of high magnitude impacts during gameplay. The Severe HAE thresholds were based on previous research by Bussey [13], while the PAV Severe HAE thresholds were derived from Ward et al. [19]. The filter classes applied to the maximum peak HAEs were Class 0 for Player #1, Class 0 for Player #2, and Class 2 for Player #3.

**Table 1:** Peak and cumulative head acceleration event metrics for confirmed concussion cases

Player	Match exposure time (m)	Max PLA *g*	Max PAA rad/s^2^	Max ∆PAV rad/s	Max RVCI rad/s	Number of HAEs	Cumulative PLA *g*	Cumulative PAA rad/s^2^	Cumulative ∆PAV rad/s
Player #1	20	36	3347	21.9	19.8	7	148.8	11,421	87.2
Player #2	42	48.4	3234	17.7	14.6	10	251.5	17,340	144.3
Player#3*	9	27.6	4089	28.5	27.3	2	35.6	5298	35.5

*Player lost consciousness

The reported maximum values in Table 1 represent the highest magnitudes recorded during the match. For Player #1 and Player #2 these values were not associated with the same HAE. In the case of Player #3, the maximum values were from the HAE that caused the loss of consciousness

For Player 1, the highest cumulative load recorded in a single match, without concussion, occurred over 43 min and included a total of 309.1 *g*, 27,534 rad/s^2^, and 140.8 rad/s for ΔPAV, with a RVCI of 147.2 rad/s, values notably higher than those recorded in the match where the player sustained a concussion.

For Player 2, the highest cumulative load in a single match without concussion was recorded over 68 min and totalled 304 *g*, 27,340 rad/s^2^, and 232.9 rad/s for ΔPAV, with an RVCI of 198.6 rad/s, again higher than the peak values observed in the match where the player sustained a concussion.

For Player 3, the highest cumulative load in a match without concussion occurred over 54 min, with a total of 117.6 *g*, 11,871 rad/s^2^, and 87.4 rad/s for ΔPAV, and an RVCI of 83 rad/s, higher than the values recorded in the match associated with their concussion.

## Discussion

This case study presents biomechanical profiles of three confirmed concussions in professional U21 academy rugby players recorded using iMGs. Each incident offers unique insights into the range of linear and angular kinematics associated with concussive HAEs, as well as angular measures (ΔPAV and the RVCI). These angular metrics explain angular burden and may offer clinical relevance beyond peak PAV alone. Of note is the variation in biomechanical profiles observed across these three players, which highlights the individual-specific nature of brain injury risk.

When comparing the three players in this study to previously published concussion cases, our findings fall within, and in some cases exceed, the ranges reported. For example, Kieffer et al. [20, 21] documented concussive HAEs in rugby players with PLA values between 24.0 *g* and 109.7 *g*, and ∆PAVs between 5.4 and 36.2 rad/s, with female players generally presenting lower values. One male player from their study sustained a concussion with a PLA of 109.7 *g* and a ∆PAV of 28.7 rad/. In American college football, Gabler et al. [6] and Cecchi et al. [5] reported concussive HAEs with PLA values of 53 *g* and 85.9 *g*, PAA values between 5200 and 8200 rad/s^2^, and PAV values of 12.1 and 44.5 rad/s, respectively. Conversely, the maximum kinematic values recorded for the three concussed players in this study do not exceed the thresholds that are relatively common in elite men’s rugby. For example, recent large-scale data from Tooby et al. [11] showed that a 50 *g* HAE occurs in 2.0% of carries and 1.2% of tackles, and a ΔPAV of 30 rad/s is seen in 2.8% of carries and 1.9% of tackles, yet none of those events in the study were associated with concussion. These findings underscore the limitation of using global thresholds alone to infer concussion risk, as many high-magnitude HAEs are tolerated without clinical symptoms.

Further, research by Allan et al. [22] investigated head kinematic variables in elite men's and women's rugby union to assess their ability to predict player removal for off-field Head Injury Assessment (HIA1) events. The study found that increases in PLA and ΔPAV were significantly associated with an increased likelihood of HIA1 removal in the men's game. Specifically, optimal thresholds for detecting HIA1 events were identified as 24.29 *g* for PLA and 14.75 rad/s for ΔPAV. However, the study also noted that high-magnitude HAEs are relatively common in elite rugby, and not all are associated with concussions, again, underscoring the challenge of relying solely on global thresholds for concussion detection.

Regarding the posture of Player #3's concussion event, both himself and the ball carrier were in an upright position. The opposing player had caught the ball off a kick, and Player #3 entered the contact at a high velocity where he could not affect an arm wrap tackle. This aligns closely with findings from both Fuller et al. [23] and Tucker et al. [24] which highlight the heightened injury risk associated with upright, non-arm tackles. Fuller et al. identified that “collisions”, defined as tackles without arm wrapping, pose a greater injury risk than tackles completed with an arm wrap, especially when players remain upright, a position that increases the likelihood of head impacts. Tucker et al. [24] further stated that Backs (Player #3’s positional group), often enter tackles at higher velocities and are therefore at increased risk for head injuries due to this.

This case study highlights the complexity of concussion diagnosis and the need for context-specific analysis that includes cumulative loading, individual susceptibility, and situational match factors. The angular metrics (PAA, PAV, ΔPAV, and RVCI) may offer more sensitivity to concussive injury than linear measures, echoing findings from both rugby and American football studies [16]. Player #3’s case, involving immediate loss of consciousness and the highest ΔPAV and RVCI values, underscores the potential clinical relevance of these angular indicators. Third, the cumulative burden of sub-concussive HAEs may also play a role in post-match concussion diagnosis [25, 26]. Players #1 and #2 were asymptomatic during the match but later diagnosed, with Player #2 having a total cumulative match load of 265.8 *g* and 17,887 rad/s^2^, and 133.8 rad/s. An important observation from Fig. 2 is that both Player #1 and Player #2 experienced back-to-back HAEs within the space of a minute, with the second impact in each sequence classified as severe. This pattern suggests a potential cumulative effect, where clustering of impacts may increase neuromechanical vulnerability and could contribute to increased risk of neurological impairment. Further research should also explore how cumulative match exposure and overall seasonal head impact load may contribute to concussion risk.

This study is the first in rugby union to report confirmed concussive HAEs that include RVCI and cumulative match exposure. These variables may help bridge the gap between iMG detected HAEs clinical outcomes, particularly when considering cumulative exposure. Ultimately, the variability in biomechanical signatures across these concussion cases underscores the individualised nature of brain injury risk. Rather than relying on a single threshold, future concussion models should consider a multi-metric, cumulative load framework, particularly for athletes involved in contact sports. These findings also support the continued development of sensor-based monitoring systems that integrate real-time thresholds, cumulative metrics, and contextual (e.g., video) data to better inform medical staff during and after matches.

## Limitations

This case study offers valuable insight into the biomechanical characteristics of confirmed concussions in professional U21 academy rugby union; however, several limitations must be considered. Most notably, the small number of confirmed concussion cases limits the generalisability of the findings. In addition, although iMGs are a validated tool for measuring head kinematics, their use is not without challenges. False negatives may occur if an impact occurs but fails to be captured by the iMG. This may be due to a trigger threshold issue, or if multiple impacts occur in quick succession during the device’s re-arming period. Furthermore, the accuracy of recorded kinematic values depends on the quality of the signal, and the fit (coupling) of the mouthguard to the upper dentition [15]. Finally, the signal filtering and transformation to the head’s CoG rely on proprietary algorithms that lack full transparency and external validation.

## Conclusion

This case study provides detailed biomechanical profiles of three confirmed concussions in professional U21 academy rugby players, captured using iMGs. By examining both peak and cumulative head kinematic data, along with angular-specific measures (ΔPAV and RVCI), this study highlights the variability in concussion mechanisms, ranging from single high-magnitude impacts to cumulative sub-concussive loading. The findings emphasise the importance of incorporating angular metrics into concussion monitoring frameworks and support the need for multi-metric approaches to better understand and predict head injury risk. These insights contribute to the growing body of evidence informing the development of real-time, data-driven tools for player welfare in contact sports.

## Data Availability

The datasets used and/or analysed during the current study are available from the corresponding author on reasonable request.

## Abbreviations

CoG: Centre of gravity
ΔPAV: Change in peak angular velocity
HAE: Head acceleration event
HIA1: Head injury assessment 1
iMG: Instrumented mouthguard
PAA: Peak angular acceleration
PAV: Peak angular velocity
PLA: Peak linear acceleration
RVCI: Rotational velocity change index
SAE: Sensor acceleration event
SRC: Sports-related concussion
